# Diaqua­bis­(4-formyl­benzoato-κ*O* ^1^)bis­(nicotinamide-κ*N* ^1^)nickel(II)

**DOI:** 10.1107/S1600536812026943

**Published:** 2012-06-20

**Authors:** Mustafa Sertçelik, Nagihan Çaylak Delibaş, Hacali Necefoğlu, Tuncer Hökelek

**Affiliations:** a Department of Chemistry, Kafkas University, 36100 Kars, Turkey; b Department of Physics, Sakarya University, 54187 Esentepe, Sakarya, Turkey; c Department of Physics, Hacettepe University, 06800 Beytepe, Ankara, Turkey

## Abstract

In the title complex, [Ni(C_8_H_5_O_3_)_2_(C_6_H_6_N_2_O)_2_(H_2_O)_2_], the Ni^II^ cation is located on an inversion center and is coordinated by two 4-formyl­benzoate (FB) anions, two nicotinamide (NA) ligands and two water mol­ecules. The four O atoms in the equatorial plane around the Ni^II^ cation form a slightly distorted square-planar arrangement, while the slightly distorted octa­hedral coordination is completed by the two N atoms of the NA ligands in the axial positions. The dihedral angle between the carboxyl­ate group and the adjacent benzene ring is 23.67 (8)°, while the pyridine and benzene rings are oriented at an angle of 89.04 (4)°. The coordinating water mol­ecule links with the carboxyl­ate group *via* an O—H⋯O hydrogen bond. In the crystal, N—H⋯O, O—H⋯O and weak C—H⋯O hydrogen bonds link the mol­ecules into a three-dimensional supra­molecular network. π–π contacts between benzene rings [centroid–centroid distance = 3.8414 (7) Å] may further stabilize the structure. A weak C—H⋯π inter­action also occurs.

## Related literature

For background to niacin, see: Krishnamachari (1974 ▶). For information on the nicotinic acid derivative *N*,*N*-diethyl­nicotinamide, see: Bigoli *et al.* (1972 ▶). For related structures, see: Aydın *et al.* (2012 ▶); Hökelek *et al.* (1996 ▶, 2009*a* ▶,*b* ▶); Hökelek & Necefoğlu (1998 ▶, 2007) ▶; Necefoğlu *et al.* (2011*a* ▶,*b* ▶). For bond-length data, see: Allen *et al.* (1987 ▶).
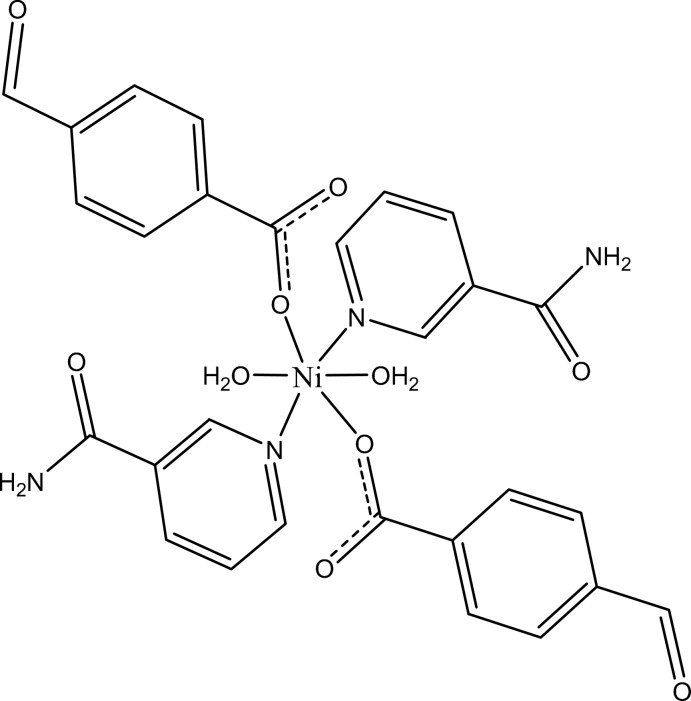


## Experimental

### 

#### Crystal data

[Ni(C_8_H_5_O_3_)_2_(C_6_H_6_N_2_O)_2_(H_2_O)_2_]
*M* *_r_* = 637.22
Triclinic, 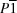

*a* = 7.7633 (2) Å
*b* = 9.8173 (3) Å
*c* = 9.8222 (3) Å
α = 78.260 (3)°
β = 71.489 (2)°
γ = 86.584 (3)°
*V* = 695.01 (4) Å^3^
*Z* = 1
Mo *K*α radiation
μ = 0.76 mm^−1^
*T* = 100 K
0.52 × 0.32 × 0.30 mm

#### Data collection

Bruker Kappa APEXII CCD area-detector diffractometer
Absorption correction: multi-scan (*SADABS*; Bruker, 2005 ▶) *T*_min_ = 0.693, *T*_max_ = 0.805
12101 measured reflections
3492 independent reflections
3429 reflections with *I* > 2σ(*I*)
*R*_int_ = 0.017

#### Refinement

*R*[*F* ^2^ > 2σ(*F* ^2^)] = 0.023
*wR*(*F* ^2^) = 0.063
*S* = 1.07
3492 reflections
216 parameters
H atoms treated by a mixture of independent and constrained refinement
Δρ_max_ = 0.40 e Å^−3^
Δρ_min_ = −0.42 e Å^−3^

### 

Data collection: *APEX2* (Bruker, 2007 ▶); cell refinement: *SAINT* (Bruker, 2007 ▶); data reduction: *SAINT*; program(s) used to solve structure: *SHELXS97* (Sheldrick, 2008 ▶); program(s) used to refine structure: *SHELXL97* (Sheldrick, 2008 ▶); molecular graphics: *ORTEP-3 for Windows* (Farrugia, 1997 ▶); software used to prepare material for publication: *WinGX* (Farrugia, 1999 ▶) and *PLATON* (Spek, 2009 ▶).

## Supplementary Material

Crystal structure: contains datablock(s) I, global. DOI: 10.1107/S1600536812026943/xu5564sup1.cif

Structure factors: contains datablock(s) I. DOI: 10.1107/S1600536812026943/xu5564Isup2.hkl

Additional supplementary materials:  crystallographic information; 3D view; checkCIF report

## Figures and Tables

**Table 1:** Hydrogen-bond geometry (Å, °) *Cg* is the centroid of the pyridine ring.

*D*—H⋯*A*	*D*—H	H⋯*A*	*D*⋯*A*	*D*—H⋯*A*
N2—H21⋯O2^i^	0.866 (18)	2.078 (18)	2.8774 (13)	153.3 (16)
N2—H22⋯O4^ii^	0.86 (2)	2.05 (2)	2.8936 (15)	166 (2)
O5—H51⋯O4^iii^	0.81 (2)	2.08 (2)	2.8628 (12)	161.1 (19)
O5—H52⋯O2^iv^	0.85 (2)	1.84 (2)	2.6634 (13)	163 (2)
C6—H6⋯O2^iii^	0.93	2.38	3.3053 (15)	172
C13—H13⋯O3^v^	0.93	2.48	3.3081 (18)	148
C4—H4⋯*Cg* ^vi^	0.93	2.74	3.6489 (14)	167

Symmetry codes: (i) 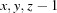; (ii) 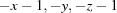; (iii) 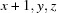; (iv) 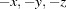; (v) 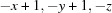; (vi) 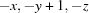.

**Fig. 1.**
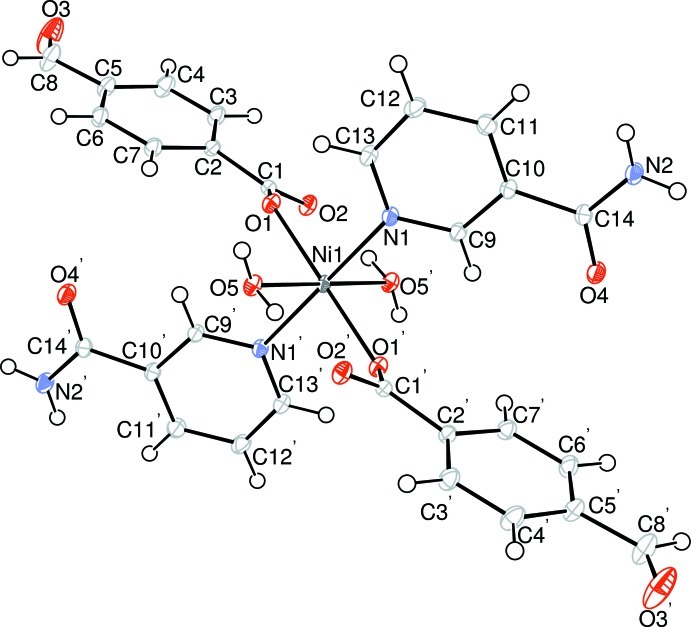
The molecular structure of the title molecule with the atom-numbering scheme. Displacement ellipsoids are drawn at the 50% probability level [symmetry code: (') -x, -y, -z].

## supplementary crystallographic information

### Comment

As a part of our ongoing investigations of transition metal complexes of nicotinamide (NA), one form of niacin (Krishnamachari, 1974), and/or the nicotinic acid derivative *N*,*N*-diethylnicotinamide (DENA), an important respiratory stimulant (Bigoli *et al.*, 1972), the title compound was synthesized and its crystal structure is reported herein.

The asymmetric unit of the title mononuclear Ni^II^ complex, (Fig. 1), contains one-half molecule. It consists of two nicotinamide (NA), two 4-formylbenzoate (FB) ligands and two coordinated water molecules, all ligands coordinating in a monodentate manner. The crystal structures of similar complexes of Cu^II^, Co^II^, Ni^II^, Mn^II^ and Zn^II^ ions, [Cu(C_7_H_5_O_2_)_2_(C_10_H_14_N_2_O)_2_] (Hökelek *et al.*, 1996), [Cu(C_7_H_4_BrO_2_)_2_(C_6_H_6_N_2_O)_2_(H_2_O)_2_] (Necefoğlu *et al.*, 2011*a*), [Co(C_6_H_6_N_2_O)_2_(C_7_H_4_NO_4_)_2_(H_2_O)_2_] (Hökelek & Necefouglu, 1998), [Co(C_9_H_9_O_2_)_2_(C_10_H_14_N_2_O)_2_(H_2_O)_2_] (Necefoğlu *et al.*, 2011*b*), [Co(C_7_H_4_IO_2_)_2_(C_6_H_6_N_2_O)_2_(H_2_O)_2_] (Aydın *et al.*, 2012), [Ni(C_7_H_4_ClO_2_)_2_(C_6_H_6_N_2_O)_2_(H_2_O)_2_] (Hökelek *et al.*, 2009*a*), [Mn(C_9_H_10_NO_2_)_2_(H_2_O)_4_].2H_2_O (Hökelek & Necefoğlu, 2007) and [Zn(C_7_H_4_BrO_2_)_2_(C_6_H_6_N_2_O)_2_(H_2_O)_2_] (Hökelek *et al.*, 2009*b*) have also been reported. In the copper(II) complex mentioned above the two benzoate ions coordinate to the Cu^II^ atom as bidentate ligands, while in the other structures all the ligands coordinate in a monodentate manner.

In the title complex, the four symmetry related O atoms (O1, O1', O5 and O5') in the equatorial plane around the Ni^II^ ion form a slightly distorted square-planar arrangement, while the slightly distorted octahedral coordination is completed by the two symmetry related N atoms of the NA ligands (N1 and N1') in the axial positions. The near equalities of the C1—O1 [1.2603 (13) Å] and C1—O2 [1.2594 (13) Å] bonds in the carboxylate group indicate delocalized bonding arrangement, rather than localized single and double bonds. The Ni—O bond lengths are 2.0650 (8) Å (for benzoate oxygens) and 2.0879 (8) Å (for water oxygens), and the Ni—N bond length is 2.0773 (9) Å, close to standard values (Allen *et al.*, 1987). The Ni atom is displaced out of the mean-plane of the carboxylate group (O1/C1/O2) by -0.5451 (1) Å. The dihedral angle between the planar carboxylate group and the adjacent benzene ring A (C2—C7) is 23.67 (8)°. The benzene A (C2—C7) and the pyridine B (N1/C9—C13) rings are oriented at a dihedral angle of A/B = 89.04 (4)°.

In the crystal, intermolecular N—H···O, O—H···O and weak C—H···O hydrogen bonds (Table 1) link the molecules into a three-dimensional supramolecular network, in which they may be effective in the stabilization of the structure. The π–π contact between the benzene rings, Cg1—Cg1^i^ [symmetry code: (i) 1 - x, 1 - y, 2 - z, where Cg1 is the centroid of the ring A (C2-C7)] may further stabilize the structure, with centroid-centroid distance of 3.8414 (7) Å]. A weak C-H···π interaction is also found in the crystal structure.

### Experimental

The title compound was prepared by the reaction of NiSO_4_.6H_2_O (1.31 g, 5 mmol) in H_2_O (25 ml) and NA (1.22 g, 10 mmol) in H_2_O (50 ml) with sodium 4-formylbenzoate (1.72 g, 10 mmol) in H_2_O (100 ml) at room temperature. The mixture was filtered and set aside to crystallize at ambient temperature for several days, giving blue single crystals.

### Refinement

Atoms H8 (for CH), H21 and H22 (for NH_2_) and H51 and H52 (for H_2_O) were located in a difference Fourier map and were refined freely. The C-bound H-atoms were positioned geometrically with C—H = 0.93 Å for aromatic H-atoms, and constrained to ride on their parent atoms, with *U*_iso_(H) = 1.2*U*_eq_(C).

### Crystal data

**Table:**

[Ni(C_8_H_5_O_3_)_2_(C_6_H_6_N_2_O)_2_(H_2_O)_2_]	*Z* = 1
*M**_r_* = 637.22	*F*(000) = 330
Triclinic, *P*1	*D*_x_ = 1.522 Mg m^−^^3^
Hall symbol: -P 1	Mo *K*α radiation, λ = 0.71073 Å
*a* = 7.7633 (2) Å	Cell parameters from 9915 reflections
*b* = 9.8173 (3) Å	θ = 2.2–28.5°
*c* = 9.8222 (3) Å	µ = 0.76 mm^−^^1^
α = 78.260 (3)°	*T* = 100 K
β = 71.489 (2)°	Block, blue
γ = 86.584 (3)°	0.52 × 0.32 × 0.30 mm
*V* = 695.01 (4) Å^3^	

### Data collection

**Table:**

Bruker Kappa APEXII CCD area-detector diffractometer	3492 independent reflections
Radiation source: fine-focus sealed tube	3429 reflections with *I* > 2σ(*I*)
Graphite monochromator	*R*_int_ = 0.017
φ and ω scans	θ_max_ = 28.5°, θ_min_ = 2.2°
Absorption correction: multi-scan (*SADABS*; Bruker, 2005)	*h* = −10→10
*T*_min_ = 0.693, *T*_max_ = 0.805	*k* = −13→11
12101 measured reflections	*l* = −13→13

### Refinement

**Table:**

Refinement on *F*^2^	Primary atom site location: structure-invariant direct methods
Least-squares matrix: full	Secondary atom site location: difference Fourier map
*R*[*F*^2^ > 2σ(*F*^2^)] = 0.023	Hydrogen site location: inferred from neighbouring sites
*wR*(*F*^2^) = 0.063	H atoms treated by a mixture of independent and constrained refinement
*S* = 1.07	*w* = 1/[σ^2^(*F*_o_^2^) + (0.0285*P*)^2^ + 0.3781*P*] where *P* = (*F*_o_^2^ + 2*F*_c_^2^)/3
3492 reflections	(Δ/σ)_max_ < 0.001
216 parameters	Δρ_max_ = 0.40 e Å^−^^3^
0 restraints	Δρ_min_ = −0.42 e Å^−^^3^

### Special details

**Table:**

Geometry. All esds (except the esd in the dihedral angle between two l.s. planes) are estimated using the full covariance matrix. The cell esds are taken into account individually in the estimation of esds in distances, angles and torsion angles; correlations between esds in cell parameters are only used when they are defined by crystal symmetry. An approximate (isotropic) treatment of cell esds is used for estimating esds involving l.s. planes.
Refinement. Refinement of F^2^ against ALL reflections. The weighted R-factor wR and goodness of fit S are based on F^2^, conventional R-factors R are based on F, with F set to zero for negative F^2^. The threshold expression of F^2^ > 2sigma(F^2^) is used only for calculating R-factors(gt) etc. and is not relevant to the choice of reflections for refinement. R-factors based on F^2^ are statistically about twice as large as those based on F, and R- factors based on ALL data will be even larger.

### Fractional atomic coordinates and isotropic or equivalent isotropic displacement parameters (Å^2^)

**Table:**

	*x*	*y*	*z*	*U*_iso_*/*U*_eq_	
Ni1	0.0000	0.0000	0.0000	0.01015 (6)	
O1	0.12386 (11)	0.17618 (8)	0.01075 (9)	0.01391 (16)	
O2	−0.11022 (11)	0.27566 (9)	0.15727 (9)	0.01583 (16)	
O3	0.49635 (15)	0.66730 (13)	0.32149 (15)	0.0408 (3)	
O4	−0.43537 (11)	0.00326 (9)	−0.33242 (9)	0.01767 (17)	
O5	0.25997 (11)	−0.08088 (9)	−0.07688 (9)	0.01421 (16)	
H51	0.342 (3)	−0.039 (2)	−0.144 (2)	0.032 (5)*	
H52	0.234 (3)	−0.148 (2)	−0.110 (2)	0.040 (5)*	
N1	−0.00291 (12)	0.09073 (10)	−0.20966 (10)	0.01250 (17)	
N2	−0.32830 (15)	0.13256 (11)	−0.55956 (11)	0.0177 (2)	
H21	−0.244 (2)	0.1839 (19)	−0.627 (2)	0.028 (4)*	
H22	−0.413 (3)	0.1004 (19)	−0.584 (2)	0.030 (4)*	
C1	0.05648 (14)	0.26130 (11)	0.09359 (12)	0.01225 (19)	
C2	0.18641 (14)	0.34931 (11)	0.12534 (12)	0.0124 (2)	
C3	0.12507 (15)	0.47036 (12)	0.18010 (13)	0.0170 (2)	
H3	0.0075	0.5014	0.1876	0.020*	
C4	0.23857 (16)	0.54394 (13)	0.22305 (14)	0.0194 (2)	
H4	0.1981	0.6249	0.2586	0.023*	
C5	0.41423 (15)	0.49603 (12)	0.21275 (13)	0.0170 (2)	
C6	0.47692 (15)	0.37788 (12)	0.15551 (13)	0.0169 (2)	
H6	0.5950	0.3476	0.1470	0.020*	
C7	0.36357 (15)	0.30452 (12)	0.11081 (13)	0.0148 (2)	
H7	0.4061	0.2259	0.0714	0.018*	
C8	0.53770 (17)	0.56908 (15)	0.26220 (16)	0.0258 (3)	
H8	0.662 (2)	0.5300 (18)	0.2443 (19)	0.028 (4)*	
C9	−0.14477 (14)	0.06781 (11)	−0.25219 (12)	0.0125 (2)	
H9	−0.2401	0.0127	−0.1855	0.015*	
C10	−0.15619 (15)	0.12233 (11)	−0.39102 (12)	0.0127 (2)	
C11	−0.01445 (16)	0.20605 (13)	−0.48952 (12)	0.0171 (2)	
H11	−0.0177	0.2445	−0.5834	0.020*	
C12	0.13220 (16)	0.23147 (13)	−0.44594 (13)	0.0182 (2)	
H12	0.2282	0.2877	−0.5099	0.022*	
C13	0.13333 (15)	0.17183 (12)	−0.30580 (12)	0.0149 (2)	
H13	0.2321	0.1885	−0.2771	0.018*	
C14	−0.31879 (15)	0.08302 (12)	−0.42590 (12)	0.0136 (2)	

### Atomic displacement parameters (Å^2^)

**Table:**

	*U*^11^	*U*^22^	*U*^33^	*U*^12^	*U*^13^	*U*^23^
Ni1	0.00887 (9)	0.01238 (10)	0.01030 (10)	−0.00088 (7)	−0.00393 (7)	−0.00292 (7)
O1	0.0129 (4)	0.0155 (4)	0.0142 (4)	−0.0020 (3)	−0.0041 (3)	−0.0043 (3)
O2	0.0111 (4)	0.0180 (4)	0.0189 (4)	−0.0010 (3)	−0.0039 (3)	−0.0057 (3)
O3	0.0243 (5)	0.0447 (7)	0.0648 (8)	−0.0018 (5)	−0.0123 (5)	−0.0383 (6)
O4	0.0159 (4)	0.0236 (4)	0.0141 (4)	−0.0064 (3)	−0.0060 (3)	−0.0012 (3)
O5	0.0107 (4)	0.0167 (4)	0.0154 (4)	−0.0011 (3)	−0.0032 (3)	−0.0045 (3)
N1	0.0119 (4)	0.0141 (4)	0.0127 (4)	−0.0009 (3)	−0.0049 (3)	−0.0032 (3)
N2	0.0178 (5)	0.0232 (5)	0.0136 (5)	−0.0063 (4)	−0.0076 (4)	−0.0010 (4)
C1	0.0130 (5)	0.0121 (5)	0.0119 (5)	−0.0017 (4)	−0.0056 (4)	0.0004 (4)
C2	0.0120 (5)	0.0128 (5)	0.0124 (5)	−0.0022 (4)	−0.0041 (4)	−0.0014 (4)
C3	0.0116 (5)	0.0160 (5)	0.0239 (6)	0.0006 (4)	−0.0052 (4)	−0.0061 (4)
C4	0.0151 (5)	0.0165 (5)	0.0281 (6)	0.0000 (4)	−0.0049 (5)	−0.0108 (5)
C5	0.0140 (5)	0.0174 (5)	0.0210 (5)	−0.0028 (4)	−0.0053 (4)	−0.0064 (4)
C6	0.0120 (5)	0.0169 (5)	0.0232 (6)	0.0007 (4)	−0.0069 (4)	−0.0050 (4)
C7	0.0133 (5)	0.0131 (5)	0.0188 (5)	0.0005 (4)	−0.0052 (4)	−0.0048 (4)
C8	0.0164 (6)	0.0290 (7)	0.0370 (7)	−0.0027 (5)	−0.0087 (5)	−0.0161 (6)
C9	0.0114 (5)	0.0139 (5)	0.0127 (5)	−0.0016 (4)	−0.0039 (4)	−0.0027 (4)
C10	0.0127 (5)	0.0139 (5)	0.0130 (5)	−0.0006 (4)	−0.0051 (4)	−0.0041 (4)
C11	0.0176 (5)	0.0212 (6)	0.0119 (5)	−0.0040 (4)	−0.0051 (4)	−0.0002 (4)
C12	0.0147 (5)	0.0214 (6)	0.0164 (5)	−0.0064 (4)	−0.0031 (4)	−0.0001 (4)
C13	0.0119 (5)	0.0168 (5)	0.0167 (5)	−0.0022 (4)	−0.0046 (4)	−0.0037 (4)
C14	0.0137 (5)	0.0157 (5)	0.0134 (5)	0.0001 (4)	−0.0056 (4)	−0.0047 (4)

### Geometric parameters (Å, º)

**Table:**

Ni1—O1	2.0650 (8)	C3—C4	1.3827 (16)
Ni1—O1^i^	2.0650 (8)	C3—H3	0.9300
Ni1—O5	2.0879 (8)	C4—H4	0.9300
Ni1—O5^i^	2.0879 (8)	C5—C4	1.3952 (16)
Ni1—N1	2.0773 (9)	C5—C8	1.4793 (16)
Ni1—N1^i^	2.0773 (9)	C6—C5	1.3865 (16)
O1—C1	1.2603 (13)	C6—C7	1.3912 (15)
O2—C1	1.2594 (13)	C6—H6	0.9300
O3—C8	1.2021 (17)	C7—H7	0.9300
O4—C14	1.2408 (14)	C8—H8	0.993 (18)
O5—H51	0.82 (2)	C9—C10	1.3885 (15)
O5—H52	0.85 (2)	C9—H9	0.9300
N1—C9	1.3409 (13)	C10—C11	1.3894 (15)
N1—C13	1.3433 (14)	C10—C14	1.4987 (15)
N2—C14	1.3278 (15)	C11—C12	1.3887 (16)
N2—H21	0.866 (18)	C11—H11	0.9300
N2—H22	0.865 (19)	C12—H12	0.9300
C2—C1	1.5081 (14)	C13—C12	1.3845 (16)
C2—C3	1.3995 (15)	C13—H13	0.9300
C2—C7	1.3907 (15)		
			
O1^i^—Ni1—O1	180.00 (4)	C3—C4—C5	119.58 (11)
O1—Ni1—O5	87.28 (3)	C3—C4—H4	120.2
O1^i^—Ni1—O5	92.72 (3)	C5—C4—H4	120.2
O1—Ni1—O5^i^	92.72 (3)	C4—C5—C8	121.24 (11)
O1^i^—Ni1—O5^i^	87.28 (3)	C6—C5—C4	120.33 (10)
O1—Ni1—N1	89.80 (3)	C6—C5—C8	118.43 (11)
O1^i^—Ni1—N1	90.20 (3)	C5—C6—C7	120.16 (10)
O1—Ni1—N1^i^	90.20 (3)	C5—C6—H6	119.9
O1^i^—Ni1—N1^i^	89.80 (3)	C7—C6—H6	119.9
O5—Ni1—O5^i^	180.00 (5)	C2—C7—C6	119.70 (10)
N1—Ni1—O5	92.75 (3)	C2—C7—H7	120.2
N1^i^—Ni1—O5	87.25 (3)	C6—C7—H7	120.2
N1—Ni1—O5^i^	87.25 (3)	O3—C8—C5	124.85 (12)
N1^i^—Ni1—O5^i^	92.75 (3)	O3—C8—H8	120.3 (10)
N1—Ni1—N1^i^	180.0	C5—C8—H8	114.8 (10)
C1—O1—Ni1	126.76 (7)	N1—C9—C10	123.16 (10)
Ni1—O5—H51	123.5 (13)	N1—C9—H9	118.4
Ni1—O5—H52	99.2 (13)	C10—C9—H9	118.4
H51—O5—H52	105.1 (18)	C9—C10—C11	118.14 (10)
C9—N1—Ni1	119.42 (7)	C9—C10—C14	117.48 (10)
C9—N1—C13	118.17 (10)	C11—C10—C14	124.36 (10)
C13—N1—Ni1	122.41 (7)	C10—C11—H11	120.5
C14—N2—H21	123.2 (12)	C12—C11—C10	119.09 (10)
C14—N2—H22	117.9 (12)	C12—C11—H11	120.5
H21—N2—H22	118.1 (16)	C11—C12—H12	120.5
O1—C1—C2	117.45 (9)	C13—C12—C11	118.97 (10)
O2—C1—O1	125.71 (10)	C13—C12—H12	120.5
O2—C1—C2	116.78 (10)	N1—C13—C12	122.46 (10)
C3—C2—C1	120.03 (10)	N1—C13—H13	118.8
C7—C2—C1	119.88 (10)	C12—C13—H13	118.8
C7—C2—C3	119.91 (10)	O4—C14—N2	122.55 (10)
C2—C3—H3	119.9	O4—C14—C10	119.75 (10)
C4—C3—C2	120.27 (10)	N2—C14—C10	117.66 (10)
C4—C3—H3	119.9		
			
O1—Ni1—N1—C9	−143.54 (8)	C1—C2—C3—C4	173.58 (11)
O1^i^—Ni1—N1—C9	36.46 (8)	C7—C2—C3—C4	−1.50 (18)
O1—Ni1—N1—C13	36.78 (9)	C1—C2—C7—C6	−172.92 (10)
O1^i^—Ni1—N1—C13	−143.22 (9)	C3—C2—C7—C6	2.17 (17)
O5—Ni1—O1—C1	−154.22 (9)	C2—C3—C4—C5	−0.60 (19)
O5^i^—Ni1—O1—C1	25.78 (9)	C6—C5—C4—C3	2.05 (19)
O5—Ni1—N1—C9	129.19 (8)	C8—C5—C4—C3	−177.91 (12)
O5^i^—Ni1—N1—C9	−50.81 (8)	C4—C5—C8—O3	4.6 (2)
O5—Ni1—N1—C13	−50.49 (9)	C6—C5—C8—O3	−175.38 (15)
O5^i^—Ni1—N1—C13	129.51 (9)	C7—C6—C5—C4	−1.38 (19)
N1—Ni1—O1—C1	113.02 (9)	C7—C6—C5—C8	178.58 (12)
N1^i^—Ni1—O1—C1	−66.98 (9)	C5—C6—C7—C2	−0.73 (18)
Ni1—O1—C1—O2	−19.24 (16)	N1—C9—C10—C11	−0.85 (17)
Ni1—O1—C1—C2	157.72 (7)	N1—C9—C10—C14	177.32 (10)
Ni1—N1—C9—C10	−178.84 (8)	C9—C10—C11—C12	0.18 (17)
C13—N1—C9—C10	0.85 (16)	C14—C10—C11—C12	−177.85 (11)
Ni1—N1—C13—C12	179.48 (9)	C9—C10—C14—O4	−0.81 (16)
C9—N1—C13—C12	−0.20 (17)	C9—C10—C14—N2	−178.60 (10)
C3—C2—C1—O1	161.91 (10)	C11—C10—C14—O4	177.24 (11)
C3—C2—C1—O2	−20.86 (15)	C11—C10—C14—N2	−0.56 (17)
C7—C2—C1—O1	−23.01 (15)	C10—C11—C12—C13	0.42 (18)
C7—C2—C1—O2	154.22 (11)	N1—C13—C12—C11	−0.43 (18)

Symmetry code: (i) −*x*, −*y*, −*z*.

### Hydrogen-bond geometry (Å, º)

Cg is the centroid of the pyridine ring.

**Table:**

*D*—H···*A*	*D*—H	H···*A*	*D*···*A*	*D*—H···*A*
N2—H21···O2^ii^	0.866 (18)	2.078 (18)	2.8774 (13)	153.3 (16)
N2—H22···O4^iii^	0.86 (2)	2.05 (2)	2.8936 (15)	166 (2)
O5—H51···O4^iv^	0.81 (2)	2.08 (2)	2.8628 (12)	161.1 (19)
O5—H52···O2^i^	0.85 (2)	1.84 (2)	2.6634 (13)	163 (2)
C6—H6···O2^iv^	0.93	2.38	3.3053 (15)	172
C13—H13···O3^v^	0.93	2.48	3.3081 (18)	148
C4—H4···*Cg*^vi^	0.93	2.74	3.6489 (14)	167

Symmetry codes: (i) −*x*, −*y*, −*z*; (ii) *x*, *y*, *z*−1; (iii) −*x*−1, −*y*, −*z*−1; (iv) *x*+1, *y*, *z*; (v) −*x*+1, −*y*+1, −*z*; (vi) −*x*, −*y*+1, −*z*.

## References

[bb1] Allen, F. H., Kennard, O., Watson, D. G., Brammer, L., Orpen, A. G. & Taylor, R. (1987). *J. Chem. Soc. Perkin Trans. 2*, pp. S1–19.

[bb2] Aydın, Ö., Çaylak Delibaş, N., Necefoğlu, H. & Hökelek, T. (2012). *Acta Cryst.* E**68**, m521–m522. 10.1107/S160053681201330X PMC3343903 22589871

[bb3] Bigoli, F., Braibanti, A., Pellinghelli, M. A. & Tiripicchio, A. (1972). *Acta Cryst.* B**28**, 962–966.

[bb4] Bruker (2005). *SADABS* Bruker AXS Inc. Madison, Wisconsin, USA.

[bb5] Bruker (2007). *APEX2* and *SAINT* Bruker AXS Inc. Madison, Wisconsin, USA.

[bb6] Farrugia, L. J. (1997). *J. Appl. Cryst.* **30**, 565.

[bb7] Farrugia, L. J. (1999). *J. Appl. Cryst.* **32**, 837–838.

[bb8] Hökelek, T., Dal, H., Tercan, B., Özbek, F. E. & Necefoğlu, H. (2009*a*). *Acta Cryst.* E**65**, m466–m467. 10.1107/S1600536809011209 PMC2968958 21582397

[bb9] Hökelek, T., Dal, H., Tercan, B., Özbek, F. E. & Necefoğlu, H. (2009*b*). *Acta Cryst.* E**65**, m607–m608. 10.1107/S1600536809015645 PMC2977639 21583825

[bb10] Hökelek, T., Gündüz, H. & Necefoğlu, H. (1996). *Acta Cryst.* C**52**, 2470–2473.

[bb11] Hökelek, T. & Necefoğlu, H. (2007). *Acta Cryst.* E**63**, m821–m823.

[bb12] Hökelek, T. & Necefoğlu, H. (1998). *Acta Cryst.* C**54**, 1242–1244.

[bb13] Krishnamachari, K. A. V. R. (1974). *Am. J. Clin. Nutr.* **27**, 108–111. 10.1093/ajcn/27.2.108 4812927

[bb14] Necefoğlu, H., Maracı, A., Özbek, F. E., Tercan, B. & Hökelek, T. (2011*b*). *Acta Cryst.* E**67**, m619–m620. 10.1107/S1600536811014188 PMC3089307 21754332

[bb15] Necefoğlu, H., Özbek, F. E., Öztürk, V., Tercan, B. & Hökelek, T. (2011*a*). *Acta Cryst.* E**67**, m900–m901. 10.1107/S1600536811021696 PMC3151863 21836889

[bb16] Sheldrick, G. M. (2008). *Acta Cryst.* A**64**, 112–122. 10.1107/S0108767307043930 18156677

[bb17] Spek, A. L. (2009). *Acta Cryst.* D**65**, 148–155. 10.1107/S090744490804362X PMC2631630 19171970

